# OIP5 Interacts with NCK2 to Mediate Human Spermatogonial Stem Cell Self-Renewal and Apoptosis through Cell Cyclins and Cycle Progression and Its Abnormality Is Correlated with Male Infertility

**DOI:** 10.34133/research.0162

**Published:** 2023-06-07

**Authors:** Yinghong Cui, Wei Chen, Li Du, Zuping He

**Affiliations:** The Key Laboratory of Model Animals and Stem Cell Biology in Hunan Province; The Research Center of Reproduction and Translational Medicine of Hunan Province, Hunan Normal University School of Medicine, Changsha 410013, Hunan, China.

## Abstract

Spermatogonial stem cells (SSCs) have important applications in both reproduction and regenerative medicine. Nevertheless, specific genes and signaling transduction pathways in mediating fate decisions of human SSCs remain elusive. Here, we have demonstrated for the first time that OIP5 (Opa interacting protein 5) controlled the self-renewal and apoptosis of human SSCs. RNA sequencing identified that NCK2 was a target for OIP5 in human SSCs, and interestingly, OIP5 could interact with NCK2 as shown by Co-IP (co-immunoprecipitation), IP-MS (mass spectrometry), and GST pulldown assays. NCK2 silencing decreased human SSC proliferation and DNA synthesis but enhanced their apoptosis. Notably, NCK2 knockdown reversed the influence of OIP5 overexpression on human SSCs. Moreover, OIP5 inhibition decreased the numbers of human SSCs at S and G2/M phases, while the levels of numerous cell cycle proteins, including cyclins A2, B1, D1, E1 and H, especially cyclin D1, were remarkably reduced. Significantly, whole-exome sequencing of 777 patients with nonobstructive azoospermia (NOA) revealed 54 single-nucleotide polymorphism mutations of the *OIP5* gene (6.95%), while the level of OIP5 protein was obviously lower in testes of NOA patients compared to fertile men. Collectively, these results implicate that OIP5 interacts with NCK2 to modulate human SSC self-renewal and apoptosis via cell cyclins and cell cycle progression and that its mutation and/or lower expression is correlated with azoospermia. As such, this study offers novel insights into molecular mechanisms underlying the fate determinations of human SSCs and the pathogenesis of NOA, and it provides new targets for treating male infertility.

## Introduction

It is particularly important to uncover molecular mechanisms controlling the fate decisions of human spermatogonial stem cell (SSCs), due to their important applications in normal reproduction and regenerative medicine. This is evidenced by the findings that SSCs have great plasticity, since these cells can differentiate into spermatozoa [1], become pluripotent embryonic stem-like cells [2], and transdifferentiate into numerous types of functional cells, including hepatocytes [3] and neurons [4] as well as prostrate, uterine, and skin epithelial cells [5]. In total, 10% to 12% of couples are infertile worldwide, and about 50% of this disease can be attributed to male infertility [6]. Notably, the prevalence of male infertility has increased by 8% from 1990 to 2017 [7]. As such, male infertility has increasingly become a serious disease affecting human health and social development. Spermatogenesis comprises 3 main processes, i.e., self-renewal (proliferation and DNA synthesis) of SSCs, meiosis of spermatocytes, and transformation of round spermatids into the elongated spermatids (spermiogenesis). SSCs are the initiating cells for spermatogenesis, and they serve as the foundation of continuous production of mature and functional spermatozoa, which is essential for male fertility. Therefore, it is of great importance to identify novel genes and their targets mediating the fate determinations of human SSCs.

SSCs can self-renew, differentiate, dedifferentiate, transdifferentiate, or die by apoptosis. The fate decisions of SSCs are preciously controlled by genes and epigenetics. Progress has been achieved in uncovering molecular mechanisms underlying rodent SSC proliferation and differentiation over the past decades. Glial-derived neurotrophic factor and retinoic acid have been regarded as 2 key factors controlling self-renewal and differentiation of mouse SSCs, respectively [8]. Mice deficient in certain genes, e.g., *DOT1L* [9], *UHRF1* [10], and *BMI1* [11], fail to maintain stemness of SSCs. Fibroblast growth factor 2 has been shown to be essential for modulating survival and proliferation of mouse SSCs via phosphatidylinositol 3-kinase/AKT and RAS/RAF/mitogen-activated protein kinase signaling pathway [12]. However, mechanisms governing human SSCs are distinct from rodent SSCs, because there are distinct differences in cell types for rodent and human SSCs, seminiferous epithelium organization, pattern of SSC differentiation, and SSC frequency [13]. Notably, specific genes and signaling pathways controlling human SSC fate decisions remain to be elucidated. This is due to difficulty in obtaining sufficient human testicular tissues from patients to isolate primary SSCs, and there is no effective culture system to expand human SSCs. We have previously established a stable human SSC line by primary human SSCs overexpressing the SV40 large T antigen [14]. Significantly, our human SSC line possesses the similar biochemical phenotype and functional attributes with human primary SSCs, and these cells could be cultured in vitro with an unlimited proliferation ability to obtain sufficient SSCs for functional and mechanistic studies of genes and small noncoding RNA [14]*.*

We have reported that p21 activated kinase 1 (PAK1) is essential for human SSC proliferation [15]. Interestingly, we have revealed that the level of OIP5 (Opa interacting protein 5) is remarkably decreased by PAK1 silencing [15], reflecting that OIP5 plays an important role in regulating human SSCs. OIP5 encodes a 25-kDa protein with a coiled-coil domain required for centromere structure formation, and it plays a crucial role in cell cycle progression. It has been shown that OIP5 can form a complex that accumulates at the G1 terminal centromere and recruits centromere protein A (CENP-A), which ensures normal chromosome segregation during cell division [16]. Holliday junction recognition protein (HJURP) can bind to OIP5 and mediates the recruitment of CENP-A by Mis18 complex [17]. CDK1 regulates HJURP phosphorylation that interacts with OIP5 [17], while M18BP1 is regulated by CDK1 phosphorylation [18]. SENP6 mediates M18BP1 deubiquitination and regulates centromeric localization of CENP-A [19]. Under abnormal condition, OIP5 is involved in a variety of diseases, including colorectal cancer [20], gastric cancer [20], lung cancer [21], bladder cancer [22], and glioblastoma [23]. However, it remains unclear regarding physiological functions and mechanisms of OIP5 in controlling human SSCs. After analysis of bioinformatics (https://www.proteinatlas.org/), we found that OIP5 was dominantly expressed in testis, especially in spermatogonia and spermatocytes, which implicates that OIP5 is involved in the fate determinations of human SSCs. In this study, we are the first to report that OIP5 mediated proliferation, DNA synthesis, and apoptosis of human SSCs via interacting with NCK2 and cell cycle progression. Moreover, we revealed that mutation or lower expression of OIP5 might be associated with nonobstructive azoospermia (NOA). As such, our study sheds a novel insight into molecular mechanisms underlying the fate determinations of human SSCs and the pathogenesis of NOA, and it offers new targets for gene therapy of male infertility.

## Results

### OIP5 is predominantly expressed in human spermatogonia and spermatocytes

**Fig. 1.**
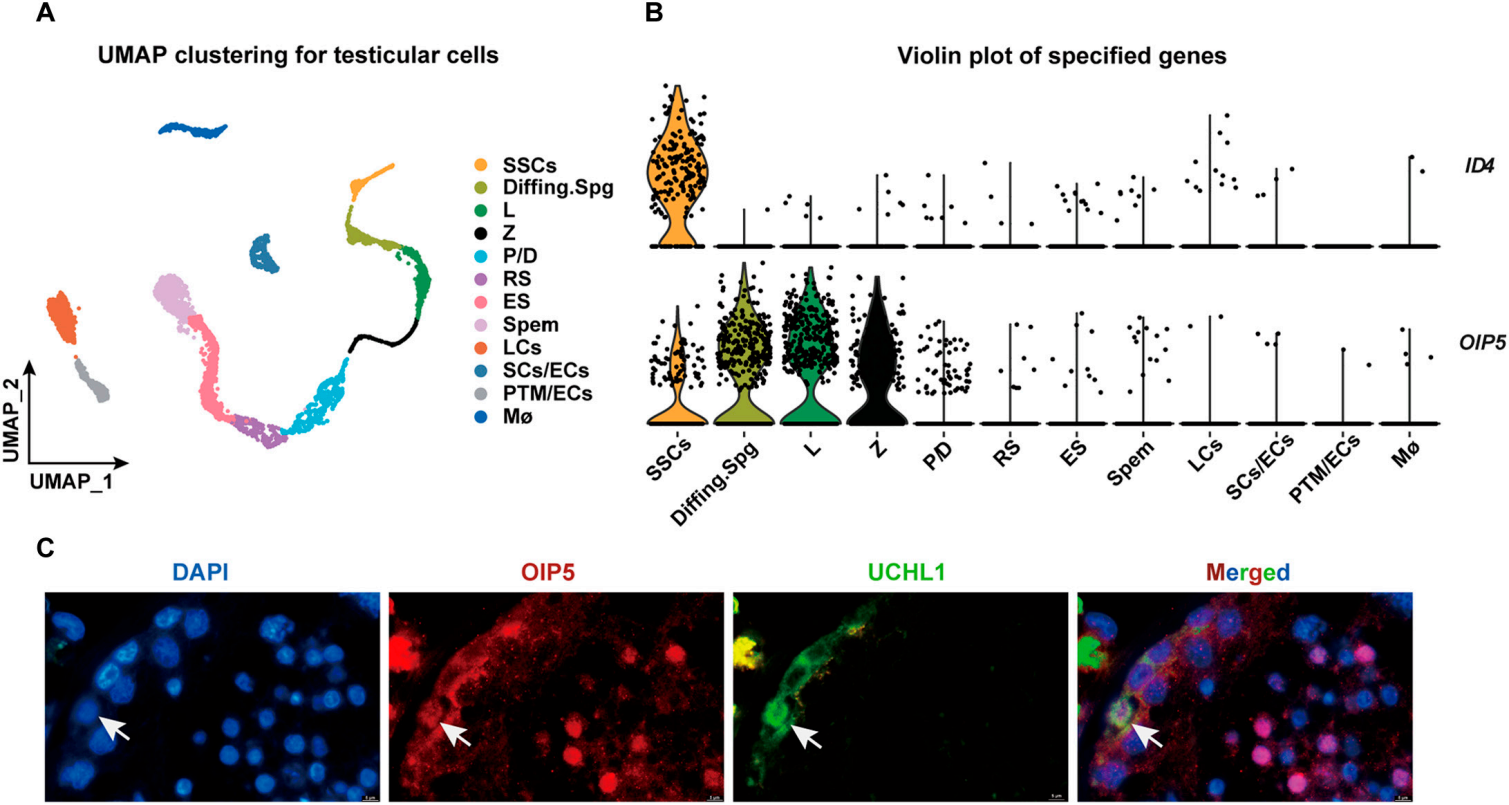
The expression of OIP5 in human testes. (A) Single-cell RNA-seq data of human testis from GSE149512 and GSE112013. (B) Violin plot showed expression pattern of *OIP5* gene in different testicular cells. (C) Double immunostaining revealed coexpression of OIP5 and UCHL1 in human testis with normal spermatogenesis. White arrows indicated OIP5-positive cells.

We first detected the expression of OIP5 in numerous kinds of human tissues. Using Human Protein Atlas (https://www.proteinatlas.org/ENSG00000104147-OIP5), we found that OIP5 protein was mainly expressed in human testis with high levels in human spermatogonia and spermatocytes (Fig S1). To demonstrate the authenticity of data from the website, we analyzed single-cell RNA sequencing datasets (GSE149512 and GSE112013) of fertile adult testis. As shown in Fig. 1A and B, *OIP5* gene was present in SSCs, differentiating spermatogonia, leptotene spermatocytes, and zygotene spermatocytes. We then used double immunostaining of UCHL1, a marker of human SSCs, and OIP5 to localize subcellular expression in human testis with normal spermatogenesis. We revealed that OIP5 was coexpressed with UCHL1 in human SSCs (arrows) (Fig. 1C) and that it was detected in spermatocytes (Fig. 1C). These results implicate that OIP5 plays an important role in regulating the mitosis and meiosis of human spermatogenesis. In this study, we focused on the role and mechanism of OIP5 in mediating fate determinations of human SSCs.

### OIP5 silencing inhibits proliferation and DNA synthesis of the human SSC line and enhances its apoptosis

**Fig. 2.**
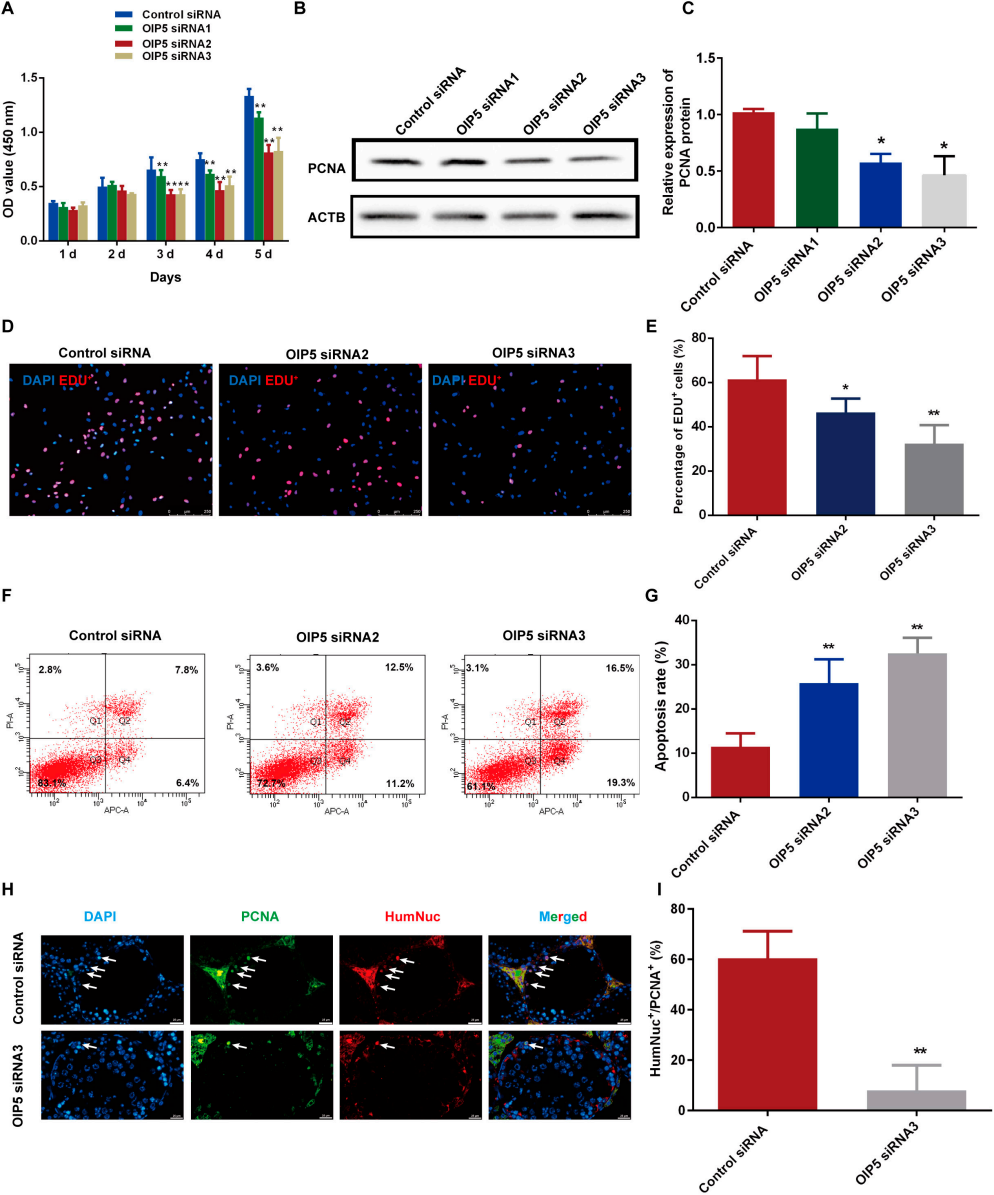
OIP5 knockdown controlled proliferation, DNA synthesis, and apoptosis of human SSC line. (A) CCK-8 assay demonstrated proliferation ability of human SSC line transfected with the control siRNA and OIP5 siRNAs. (B and C) The expression of PCNA protein in human SSC line transfected with the control siRNA and OIP5 siRNAs. (D and E) The percentages of EDU-positive cells in human SSC line transfected with the control siRNA and OIP5 siRNAs. (F and G) Flow cytometry detected percentages of apoptotic cells in human SSC line transfected with the control siRNA and OIP5 siRNAs. (H and I) Xenotransplantation of human SSC line into mice to evaluate the function of OIP5 in vivo. White arrow showed the positive staining. Notes: * indicated *P* < 0.05; ** denoted *P* < 0.01.

We have previously established the first human SSC line worldwide [14], and we checked the identity of this human SSC line. Immunocytochemistry (Fig. S2A) and reverse transcription polymerase chain reaction (RT-PCR) (Fig. S2B) showed that a number of markers for human primary SSC were detected in the human SSC line, including CD90, GPR125, GFRA1, PLZF, UCHL1, MAGEA4, and RET, reflecting that this human SSC line was indeed human primary SSCs phenotypically. Three small interfering RNAs (siRNAs) against OIP5 were used to silence OIP5 expression to explore its impact on the fate decisions of human SSCs. Our quantitative real-time PCR (qPCR) and Western blots showed that the expression levels of OIP5 were significantly decreased by OIP5 siRNA2 and OIP5 siRNA3 (Fig. S3). Cell counting kit-8 (CCK-8) illustrated that the number of human SSC line was remarkably reduced by OIP5 siRNA2 and OIP5 siRNA3 at days 3 to 5 (Fig. 2A). Meanwhile, the expression level of PCNA (Fig. 2B and C), a hallmark for cellular proliferation, and percentages of EDU-positive cells (Fig. 2D and E) were decreased by OIP5 siRNA2 and OIP5 siRNA3 in human SSC line. In contrast, OIP5 overexpression resulted in the increases in PCNA level (Fig. S4B and C) and DNA synthesis (Fig. S4D and E). Furthermore, we detected the influence of OIP5 knockdown on apoptosis of human SSC line. As shown in Fig. 2F and G, the late and early apoptosis of human SSC line was dramatically enhanced by OIP5 siRNA2 and OIP5 siRNA3 after transfection for 72 h. By contrast, OIP5 overexpression inhibited cell apoptosis of human SSC line (Fig. S4F and G).

To further clarify the function of OIP5 in mediating human SSCs in vivo, male mouse germ cells were depleted by busulfan, and xenotransplantation of human SSC line treatment with OIP5 siRNA3 or control siRNA into mouse seminiferous tubules was performed. Two months after xenotransplantation, anti-PCNA antibody and anti-human nuclear antigen (HumNuc) were utilized to detect the survival and proliferation of the transplanted cells. We found that HumNuc^+^ and PCNA^+^ cells in the testes with OIP5 siRNA3-transfected cells were obviously fewer than those in control siRNA (Fig. 2H and I). As our human SSC line expresses SV40 stably, we examined the colocalization of SV40 and PCNA. Consistently, we observed fewer SV40^+^/PCNA^+^ cells by OIP5 siRNA3-transfection compared with the control siRNA (Fig. S5). Taken together, these data suggest that OIP5 enhances proliferation & DNA synthesis and inhibits cell apoptosis of the human SSC line.

### NCK2 is a target of OIP5 and they interact with each other in human SSCs

**Fig. 3.**
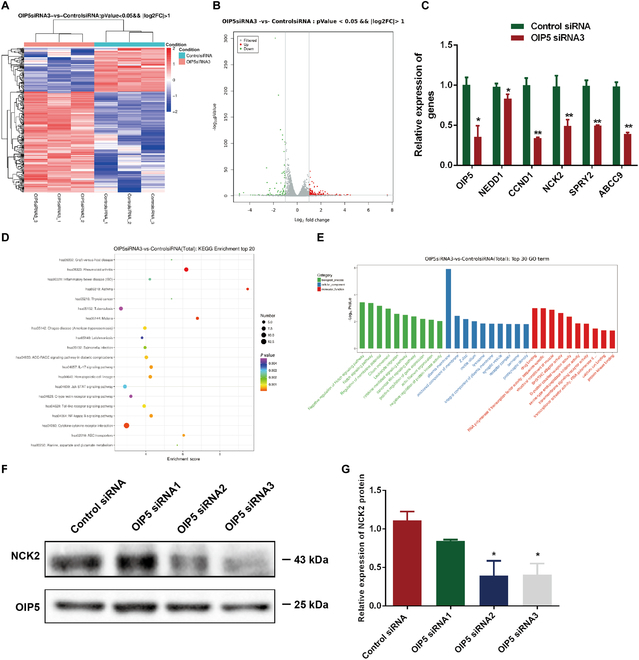
NCK2 was identified as a target of OIP5 in human SSCs. (A) Hierarchical clustering showed the DEGs in human SSC line transfected with control siRNA and OIP5 siRNA3. Notes: Red color represented relatively high expression level of genes, and blue color indicated relatively low expression level of genes. (B) Volcano plot illustrated the DEGs in human SSC line between control siRNA and OIP5 siRNA3. (C) qPCR revealed the different expression of *OIP5*, *NCK2*, *NEDD1*, *CCND1*, *SPRY2*, and *ABCC9*. (D) Pathway analysis of the DEGs by KEGG. The items with larger bubbles contained more DEGs. The bubble color changed from purple-blue-green-red, which indicated the smaller the enrichment *P* value. (E) GO analysis illustrated the top 30 enrichment functional terms. (F and G) Western blots showed that NCK2 level was decreased by OIP5 siRNA2 and OIP5 siRNA3. * indicated *P* < 0.05; ** denoted *P* < 0.01.

To clarify the targets of OIP5 in human SSCs, we conducted RNA sequencing to compare global transcription profiles in human SSC line transfected with OIP5 siRNA3 and control siRNA. About 20,030 genes were detected in the human SSC line, and there were 275 differentially expressed genes (DEGs) between OIP5 siRNA3 and the control siRNA. Cluster map and volcano lot of the DEGs illustrated that 192 genes were upregulated whereas 83 genes were downregulated by OIP5 siRNA3 in the human SSC line (Fig. 3A and B). Several genes identified by RNA sequencing data, including *OIP5*, *NCK2*, *NEDD1*, *CCND1*, *SPRY2*, and *ABCC9*, were selected for verification by qPCR. Our qPCR showed that changes of those genes were consistent with RNA sequencing (Fig. 3C). Utilizing gene ontology (GO) and Kyoto Encyclopedia of Genes and Genomes (KEGG) analyses, we found that the DEGs were mainly enriched in biological processes and signal pathways related to proliferation and apoptosis (Fig. 3D and E). NCK2 is a cofactor of receptor protein tyrosine kinases that contain 3 SH3 domains and 1 SH2 domain, and it is expressed in various kinds of tissues. Interestingly, we found that the expression level of NCK2 protein was reduced by OIP5 siRNA2 and OIP5 siRNA3 (Fig. 3F and G).

**Fig. 4.**
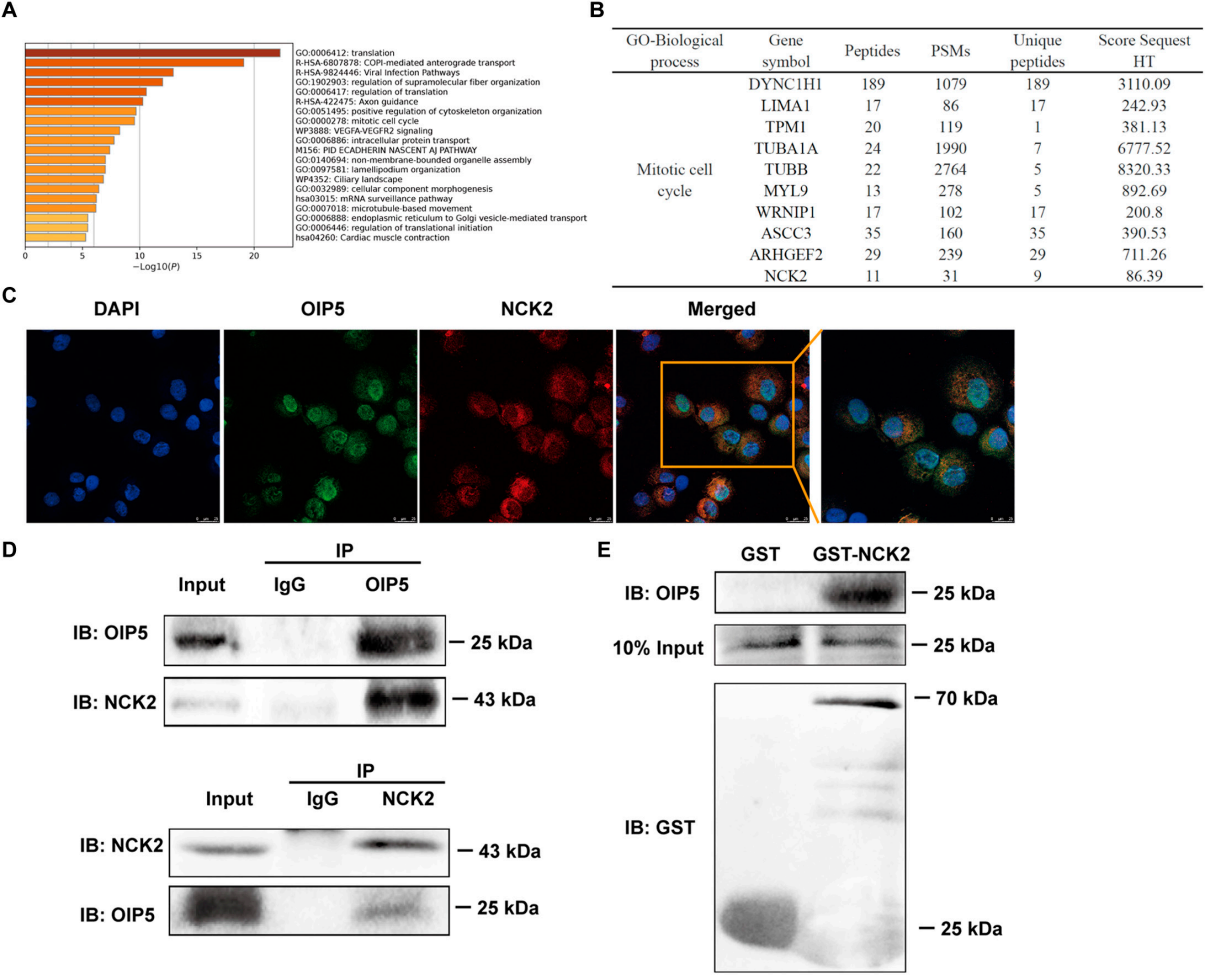
The interaction between OIP5 and NCK2 in human SSCs. (A) GO analysis for the identified proteins illustrated the top enrichment terms. (B) Gene list in biological processes of mitotic cell cycle pursuant to GO analysis. (C) Representative images of double immunostaining for OIP5 and NCK2 in human SSC line. (D) Co-IP demonstrated that OIP5 was interacted with NCK2 in human SSC line. (E) GST pulldown assay illustrated the interaction between OIP5 and NCK2 in human SSC line.

To further demonstrate the interaction between OIP5 and NCK2 in human SSCs, the Co-IP (co-immunoprecipitation), IP-MS (mass spectrometry), and glutathione S-transferase (GST) pulldown assays were employed in this study, and we identified 694 proteins with high confidence. GO analysis revealed that these proteins were enriched in the biological processes, including translation, regulation of supramolecular fiber organization, positive regulation of cytoskeleton organization, mitotic cell cycle, and intracellular protein transport (Fig. 4A). Among mitotic cell cycle regulation, NCK2, DYNCIH1, TUBA1A, and TUBB were involved in this process (Fig. 4B). Combined with our RNA-sequencing (RNA-seq) data, we speculated that NCK2 might be interacted with OIP5 directly. Next, we analyzed the mass spectrogram (MS) of NCK2 (Fig. S6). Double immunostaining illustrated that OIP5 (nuclei, red fluorescence) and NCK2 (cytoplasm, green fluorescence) were coexpressed in human SSC line (Fig. 4C). Notably, our Co-IP assay revealed that OIP5 could pull down NCK2 (Fig. 4D, upper panel) and that NCK2 could bind to OIP5 (Fig. 4D, lower panel). We then purified the expression of NCK2 to detect the direct interaction of NCK2 and OIP5 in human SSCs. Compared with the GST group, GST-NCK2 could pull down OIP5 in human SSC line (Fig. 4E). Considered together, these data implicate that NCK2 is a target of OIP5 in human SSCs and that OIP5 can interact with NCK2 in these cells.

### NCK2 silencing decreases human SSC proliferation and DNA synthesis and enhances their apoptosis

**Fig. 5.**
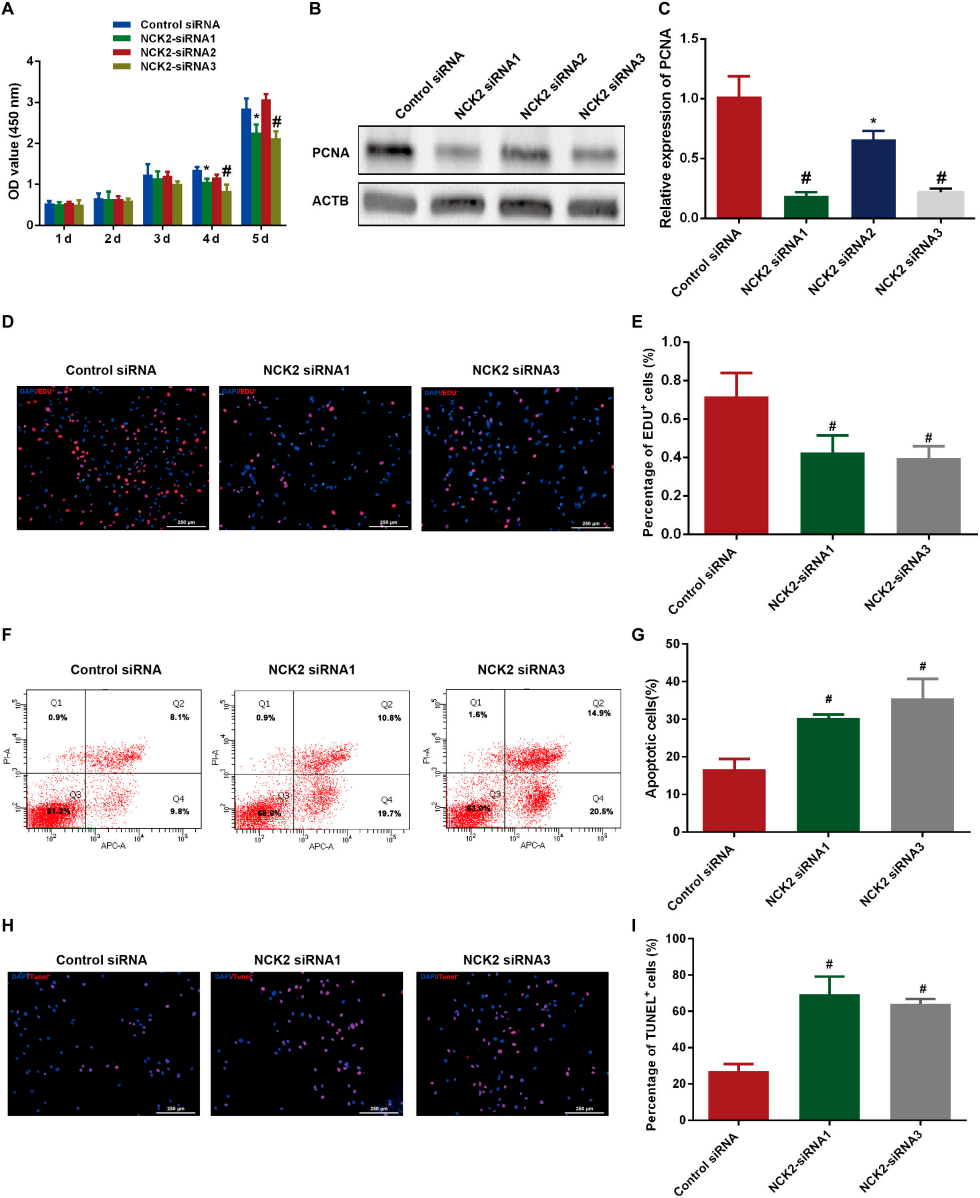
NCK2 silencing suppressed proliferation and enhanced apoptosis of human SSC line. (A) CCK-8 assay demonstrated proliferation of human SSC line transfected with the control siRNA and NCK2 siRNAs. (B and C) The expression level of PCNA protein in human SSC line transfected with the control siRNA and NCK2 siRNAs. (D and E) The percentages of EDU-positive cells in human SSC line transfected with the control siRNA and NCK2 siRNAs. (F and G) Flow cytometry detected the percentages of apoptosis cells in human SSC line treatment with the control siRNA and NCK2 siRNAs. (H and I) TUNEL assay evaluated apoptotic cells in human SSC line when NCK2 was knocked down. * indicated *P* < 0.05; # denoted *P* < 0.01.

To examine the function of NCK2 in regulating the proliferation and DNA synthesis of human SSC line, 3 NCK2 small interference RNAs (siRNAs) were designed and employed in this study. As shown in Fig. S7, the level of NCK2 was diminished by NCK siRNA1 and NCK siRNA3. CCK-8 assay indicated that both NCK2 siRNA1 and siRNA3 reduced the numbers of human SSC line after transfection of siRNAs for 4 to 5 d (Fig. 5A). As expected, PCNA expression level was decreased in the human SSC line after NCK2 siRNA1 and siRNA3 transfection (Fig. 5B and C). EDU incorporation assay illustrated that NCK2 knockdown reduced EDU-positive cells of human SSC line (Figure 5D and E). Furthermore, flow cytometry (Fig. 5F and G) and TUNEL (3,3’diaminobenzidine tetrahydrochloride) (Fig. 5H and I) assays demonstrated that apoptotic cells and TUNEL-positive cells were enhanced in the human SSC line by NCK2 silencing. Collectively, our observations indicate that NCK2 silencing leads to reduction of human SSC proliferation and DNA synthesis and an increase in cell apoptosis.

### NCK2 knockdown reverses the effect of OIP5 overexpression on human SSCs

**Fig. 6.**
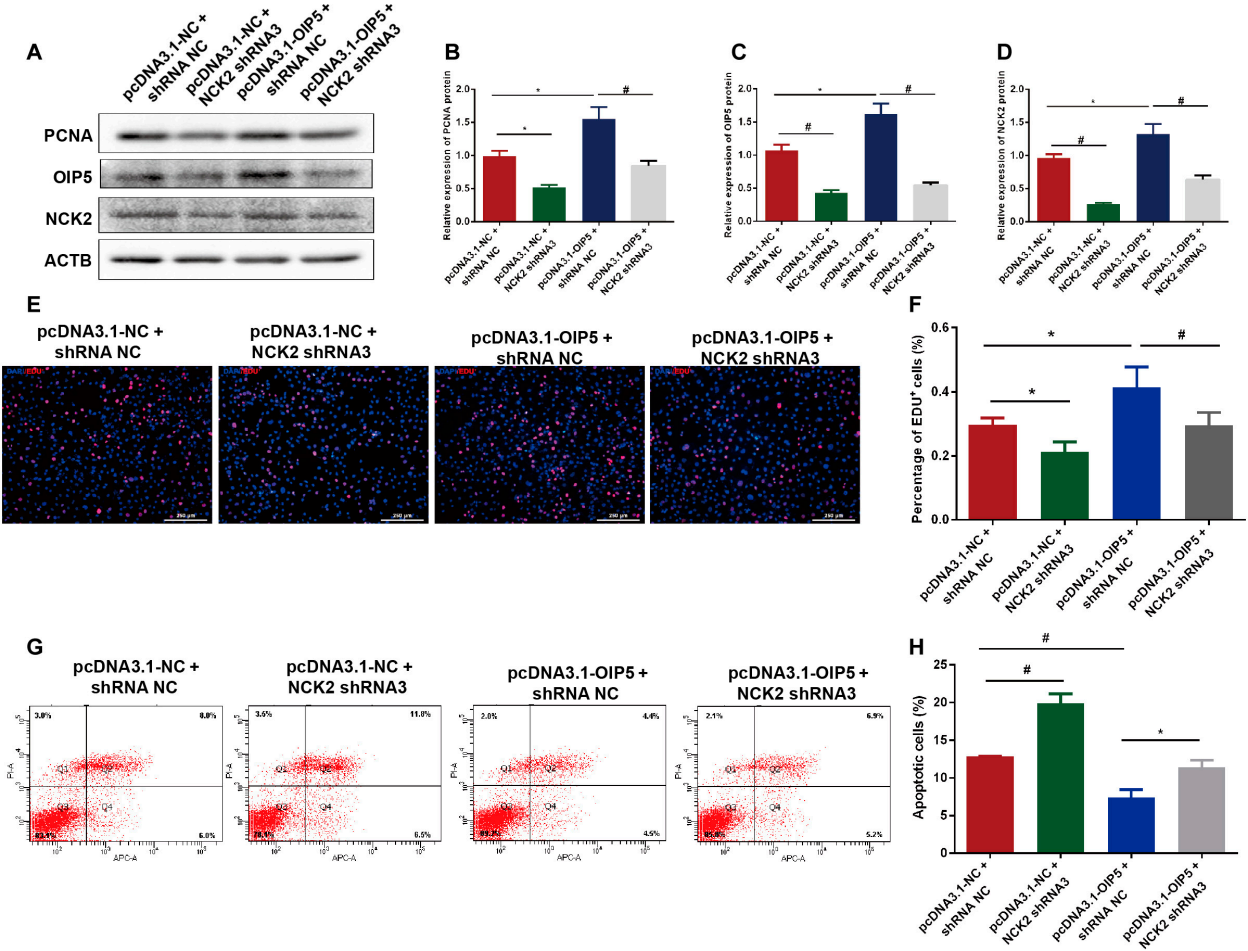
NCK2 silencing reversed the effect of OIP5 overexpression on human SSC line. (A to D) The expression of PCNA, OIP5, and NCK2 in human SSC line cotransfected with pcDNA3.1-OIP5 and NCK2 shRNA3. (E and F) The percentages of EDU-positive cells in human SSC line cotransfected with pcDNA3.1-OIP5 and NCK2 shRNA3. (G and H) Flow cytometry measured the percentages of apoptosis in human SSC line cotransfected with pcDNA3.1-OIP5 and NCK2 shRNA3. * indicated *P* < 0.05; # indicated *P* < 0.01.

To further clarify the association of NCK2 and OIP5, human SSC line was cotransfected with NCK2 shRNA (Table S1) and pcDNA3.1-OIP5. We constructed 3 NCK2 shRNAs and found that NCK2 shRNA2 and shRNA3 could significantly decrease NCK2 expression level (Fig. S8). NCK2 shRNA3 was selected to be cotransfected with pcDNA3.1-OIP5 into the human SSC line. As shown in Fig. 6A to D, NCK2 shRNA3 reduced the levels of PCNA, OIP5, and NCK2, which were enhanced by pcDNA3.1-OIP5. Consistent with these results, NCK2 shRNA3 and pcDNA3.1-OIP5 cotransfection diminished the percentages of EDU-positive cells of human SSC line compared to OIP5 overexpression (Fig. 6E and F). Moreover, apoptotic cells of human SSC line were increased by NCK2 shRNA3 and pcDNA3.1-OIP5 (Fig. 6G and H). Considered together, these data further implicate that NCK2 acts as an important downstream target of OIP5 in regulating human SSC proliferation, DNA synthesis, and apoptosis.

### OIP5 is involved in mediating cell cycle progression G1/G2/M phases of human SSCs

**Fig. 7.**
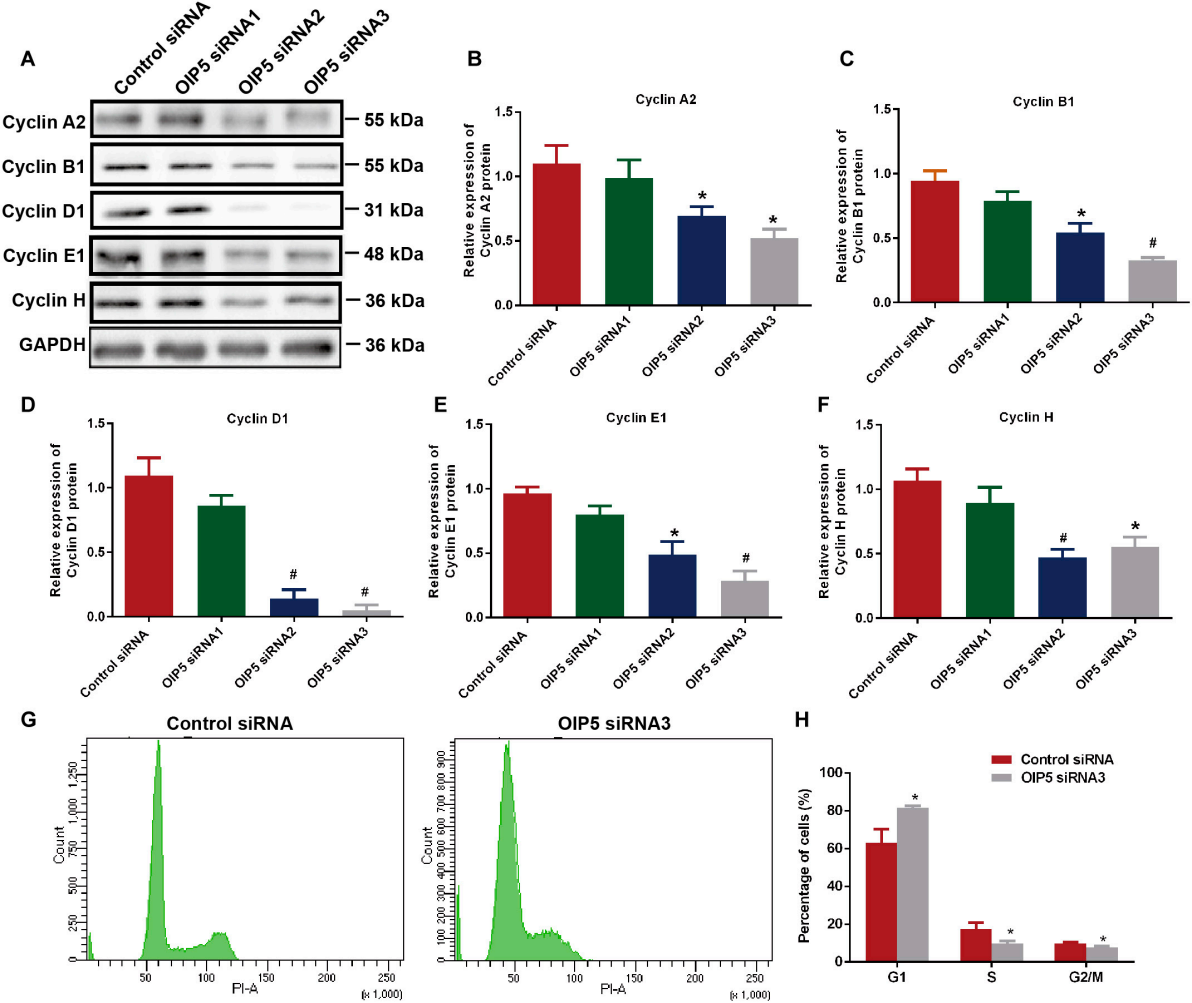
The effect of OIP5 on cell cyclins and cell cycle progression of human SSC line. (A to F) The expression of Cyclin A2, Cyclin B1, Cyclin D1, Cyclin E1, and Cyclin H in human SSC line transfected with OIP5 siRNAs. (G and H) PI staining and flow cytometry were used to detect cell cycle progression of human SSC line transfected with OIP5 siRNAs. * indicated *P* < 0.05; # indicated *P* < 0.01.

OIP5 is involved in the recruitment of CENP-A via an interaction with HJURP [17,24], and thus we asked whether OIP5 controlled the procession of cell cycles of human SSCs. To answer this question, we detected a number of cell cycle proteins in human SSC line when OIP5 was knocked down. As shown in Fig. 7A to F, OIP5 siRNA2 and siRNA3 could decrease the expression levels of numerous cell cyclins, including Cyclins A2, B1, D1, E1, and H. Especially, Cyclin D1 level was remarkably reduced by OIP5 siRNA2 and siRNA3, which was consistent with our RNA-seq data demonstrating that *Cyclin D1* was one of the most significantly down-regulated genes (fold changes: −3.57) by OIP5 siRNA3 (Fig. 3A to C). Therefore, we further asked whether OIP5 regulated G1/S transition of human SSCs. We checked cell cycle procession by flow cytometry. Interestingly, we found that cells of the human SSC line at G1 phase was increased by OIP5 siRNA3, whereas cells at S phase and G2/M phases were decreased by OIP5 silencing (Fig. 7G and H).

### OIP5 mutations and/or lower levels are associated with NOA

We finally probed whether OIP5 was associated with male infertility. We collected whole blood from 777 NOA patients, and whole-exome sequencing (WES) was performed to identify mutations of *OIP5* in NOA patients. There were 54 cases of *OIP5* gene single-nucleotide polymorphism mutations, including 1 homozygous mutation (c.494 C>T, p.S165F) and 53 heterozygous mutations, which accounted for 6.95% of *OIP5* gene mutations. Among 54 *OIP5* mutations, 53 were at exons and they were described as follows: 30 *OIP5* mutations were at exon3: c.494C>T (p.S165F), while 19 *OIP5* mutations were at exon1: c.164T>G (p.L55R); there were 2 *OIP5* mutations at exon5: c.658G>T (p.V220L), 1 *OIP5* mutation at exon1: c.77G>A (p.R26K), and 1 *OIP5* mutations at exon1: c.110T>C (p.M37T). Another *OIP5* mutation was found at the variable shear region (exon5: c.595-8C>T) (Table S2). We also predicted the potential harmfulness of mutation sites. As shown in Table S2, some variations of *OIP5* were found to be damaged, especially c.110T>C (p.M37T).

**Fig. 8.**
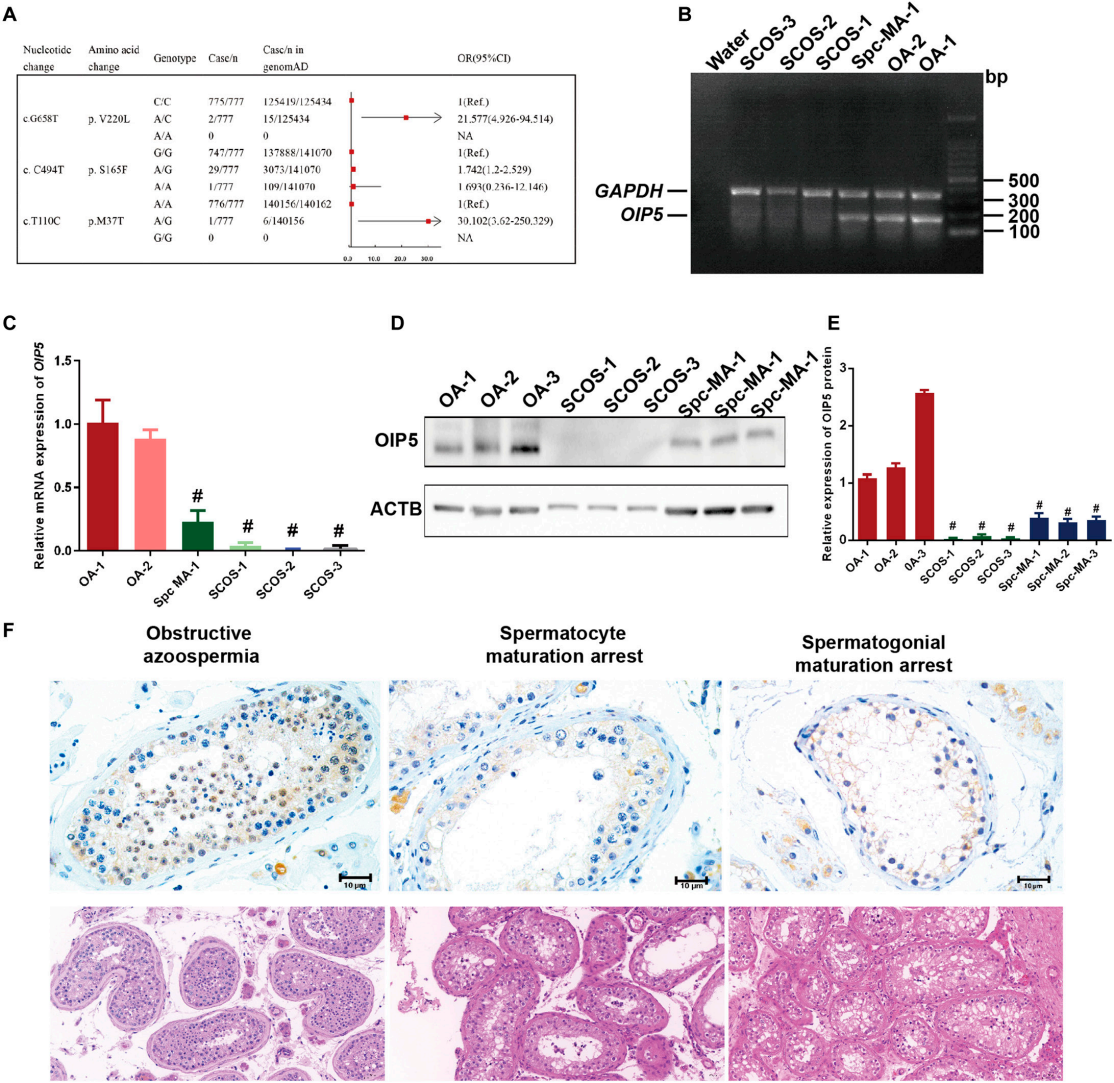
OIP5 mutations in NOA patients and OIP5 expression in human testis tissues with spermatogenesis disorders. (A) WES identified a number of variations of OIP5 and logistic regression was used to analyze the risk of variations. (B) RT-PCR analyzed the different expression of *OIP5* in testis from NOA patients. (C) qPCR analysis of *OIP5* level in testis from NOA patients. (D and E) Western blots showed the expression levels of OIP5 protein in NOA patients. (F) Immunohistochemistry revealed expression levels of OIP5 in human testis with normal spermatogenesis or spermatogenesis disorders. # indicated *P* < 0.01.

Interestingly, we performed chi-square test on *OIP5* genotype frequency and then conducted logistic regression analysis on the sites with statistical differences to clarify the correlation between *OIP5* mutations and NOA. We found that rs547051997 (χ^2^ = 34.542, *P* < 0.0001), rs560529830 (χ^2^ = 24.085, *P* = 0.038), and rs2047945082 (χ^2^ = 84.168, *P* = 0.012) of *OIP5* were statistically significant (Fig. 8A). Furthermore, we revealed that genotype frequency and allele frequency of rs547051997, rs560529830, and rs2047945082 loci of *OIP5* in NOA patients were statistically higher than those in normal population from gnomAD. These data implicate that variations of *OIP5* at the above loci were positively related to risk of NOA patients.

We also measured the expression levels of OIP5 in testis tissues from NOA patients and obstructive azoospermia patients with normal spermatogenesis. RT-PCR and qPCR demonstrated the level of *OIP5* mRNA was significantly lower in spermatocyte maturation arrest (Spc MA) and Sertoli-cell-only syndrome (SCOS) than obstructive azoospermia patients (Fig. 8B and C). Western blots (Fig. 8D and E) and immunohistochemistry (Fig. 8F) further demonstrated that OIP5 protein amount was decreased in Spc MA and SCOS compared to the control fertile men. Taken together, these results imply that OIP5 aberrant expression may contribute to spermatogenesis failure.

## Discussion

SSC is essential for normal reproduction as they can self-renew to maintain the stem cell population and differentiate into spermatocytes to produce functional spermatids [25]. Therefore, it is important to elucidate molecular mechanisms governing self-renewal (proliferation and DNA synthesis) and apoptosis of human SSCs. In the present study, we have identified a novel mechanism by OIP5 interaction with NCK2 and cell cyclins to regulate fate decisions of human SSCs. We have demonstrated that OIP5 silencing inhibited proliferation and DNA synthesis of the human SSC line and that it enhanced apoptosis of these cells via the interaction with NCK2 and mediating cell cycle procession. More importantly, we have revealed that OIP5 mutation and/or abnormal expression may be related to male infertility.

It has been reported that OIP5 induces papillary renal carcinoma cell proliferation [26]. In glioblastoma, cells exhibit obvious reduction of division when OIP5 is knocked down, whereas its recovery enhances proliferation ability [27]. Consistent with the effect of OIP5 on cancer, we have demonstrated that OIP5 knockdown significantly suppressed proliferation and DNA synthesis of human SSCs and increased their apoptosis. In glioblastoma, OIP5 depletion has no effect on apoptosis, which may be due to the different functions of OIP5 in different cell types. The proliferation affected by human SSCs OIP5 is probably due to its regulation of cell cyclins and cell cycle progression. OIP5 level has been shown to be enhanced at G1 phase [28], and it is localized to chromosomes at postmitotic stage [28]. Stable binding of OIP5 to centromeres is essential for recruitment of CENP-A [16,24,29]. These findings suggest that OIP5 is involved in regulation of cell cyclin proteins. We found that cell numbers of human SSCs at G1 phase were increased significantly by OIP5 silencing, and notably, the cells at S phase and G2/M phases were remarkably decreased by OIP5 knockdown. Significantly, a number of cell cyclins, including Cyclins A2, B1, D1, E1, and H, were reduced by OIP5 siRNAs. These results implicate that OIP5 controls human SSC proliferation and DNA synthesis through mediating cell cycle procession and cell cyclins, especially Cyclin D1, as demonstrated by our RNA interference and RNA-seq.

Centromere protein U (CENPU) has been shown to accelerate the G1/S transition of hepatocellular carcinoma cell proliferation by interacting with E2F6 and affecting transcription of E2F1 [29]. The altered genes by CENPN knockdown are enriched in pathways related to cell cycle regulation [30]. Furthermore, CENPN can interact with AKT and enhances its phosphorylation at S473, which promotes cell proliferation and cell cycle progressing of nasopharyngeal carcinoma cells [30]. To unveil new mechanism of OIP5 in regulating human SSCs, we conducted RNA-seq to screen DEGs after OIP5 knock down. Significantly, our RNA-seq analysis revealed that *NCK2* was downregulated in the human SSC line by OIP5 silencing, suggesting that NCK2 is a target of OIP5 in human SSCs. NCK2 can recruit proteins to mediate receptor protein tyrosine kinases [31], and it contains a C-terminal SH2 domain and 3 N-terminal SH3 domains with mediation [32,33]. NCK2 has been shown to control cytoskeletal organization [34]. In this study, we observed that NCK2 knockdown led to decreases in the proliferation and DNA synthesis of human SSCs and an increase in the apoptosis of these cells. Consistent with our results, NCK2 promotes the proliferation of human primary melanoma by modulating phosphatase activities [35]. Interestingly, we have revealed an interaction between NCK2 and OIP5 by Co-IP, IP-MS, and GST pull-down assays showing that OIP5 could pull down NCK2 and NCK2 was able to bind to OIP5. Moreover, we found that OIP5 silencing decreased the expression level of NCK2 in human SSC line. We also found that NCK2 knockdown could reverse the effect of OIP5 overexpression on proliferation and apoptosis of human SSC line. Considered together, our results implicate that NCK2 functions as a downstream target of OIP5 in regulating fate decisions of human SSCs.

We have previously reported that PAK1 stimulates human SSC proliferation and DNA synthesis [15]. Using RNA-seq, we have found that the expression level of OIP5 is decreased by PAK1 knockdown [15]. PAK1 belongs to serine/threonine p21-activating kinases, which play important roles in mediating cytoskeleton reorganization and nuclear signaling [36]. It would be interesting to further explore the association among OIP5, NCK2, and PAK1 to regulate fate determinations of human SSCs. Significantly, we have identified a number of variations of *OIP5* in NOA patients, and the expression level of OIP5 was significantly lower in NOA patients with spermatocyte maturation arrest, spermatogonial maturation arrest, or Sertoli-cell-only syndrome (SOCS). These results indicate that OIP5 abnormality might be correlated with spermatogenesis failure.

**Fig. 9.**
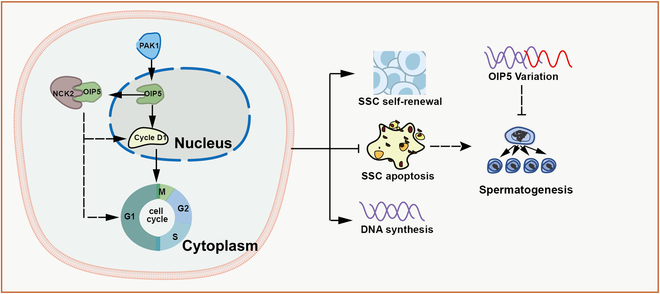
Schematic diagram illustrated the function and mechanism of OIP5 in regulating fate determinations of human SSCs as well as the association between OIP5 abnormality and spermatogenesis failure. OIP5 was regulated by PAK1, and NCK2 was a target of OIP5 in human SSCs. OIP5 could interact with NCK2 and mediated cycle cyclins and cell cycle progression at different phases to control proliferation, DNA synthesis, and apoptosis of human SSCs. The mutation and/or lower expression of OIP5 resulted in spermatogenesis failure and NOA.

In summary, we have demonstrated, for the first time, that OIP5 is primarily expressed in human SSCs and spermatocytes and that OIP5 mediates proliferation, DNA synthesis, and apoptosis of human SSCs via regulating cell cyclins and cell cycle progression. We have identified that NCK2 is an important target of OIP5 in human SSCs, and interestingly, OIP5 could interact with NCK2 in these cells. We have further revealed that mutations and lower expression of OIP5 might be correlated with NOA. This study thus provides a new molecular regulatory mechanism underlying fate determinations of human SSCs (Fig. 9), and it could provide novel targets for gene therapy of male infertility.

## Materials and Methods

### Cell culture and cell transfection

Human SSC line was established by our group using the method described previously [14]. This cell line was cultured with Dulbecco's modified Eagle's medium/F12 by addition of 10% fetal bovine serum (Gibco, 10099-141) and 100 units/ml penicillin streptomycin (Gibco, 15140-122) at 34 °C in a 5% CO_2_ incubator.

For siRNA transfection, human SSC line was plated onto 6-well plates. The siRNAs targeting different genes were synthesized by Genepharma (Suzhou, China), and siRNA sequences were shown in Table S3. Cells were transfected with 100 nM siRNAs via Lipofectamine 3000 regent (Cat No. L3000015, Invitrogen, CA, USA) pursuant to manufacturer’s instruction. These cells were collected at 24 h after siRNA transfection for gene determination, while they were harvested at 48 h after siRNA transfection for protein analyses.

### Obtainment of human testis tissues

Human testicular tissues used in this study were obtained from 9 patients at 22 to 65 years old, and these patients underwent microdissection testicular sperm extraction (m-TESE) or castration therapy for prostate cancer at Hunan Cancer Hospital or the Third Xiangya Hospital of Central South University. The tissues were washed 3 times with phosphate buffered saline (PBS) containing 4% streptomycin and penicillin, and they were fixed in Bouin's fixative solution for immunohistochemistry or stored in liquid nitrogen for protein or RNA extraction. This study was approved by The Ethical Review Committee of Hunan Normal University. Patients agreed that these tissues were to be used for scientific research only and they signed the informed consent forms.

### Immunocytochemistry

To detect the expression of SSC markers in the human SSC line, cells were collected with PBS for cytospin. The cells were fixed with 4% paraformaldehyde for 10 min. After being washed 3 times with PBS, cells were permeabilized with 0.25% Triton X-100 in PBS for 10 min, and 5% bovine serum albumin was utilized for blocking at room temperature (RT) for 1 h. Cells were incubated with primary antibodies (Table S4) at RT for 1 h or 4 °C overnight. Next, cells were washed with PBS, and they were incubated with immunoglobulin G secondary antibodies labeled by Alexa Fluor 594 (Table S5). DAPI (4′,6-diamidino-2-phenylindole) was used to visualize cell nuclei, and immunostaining was observed under a fluorescence microscope (Leica, DM3000, Germany).

### Immunohistochemistry

Testicular tissue sections were incubated at 65 °C for 90 min and dewaxed with turpentine, and dehydration with alcohol of different gradients were carried out. Sections were treated with 0.01 M citrate buffer (pH 6.0) and boiled for 10 min in a microwave oven. Next, 3% H_2_O_2_ was employed to block inactivate endogenous peroxidase at RT for 10 min. After being washed with PBS 3 times, blocking of the sections by 5% bovine serum albumin was performed, and then the slides were incubated with primary antibodies **(**Table S4) at RT for 1 h or 4 °C overnight.

For 3,3’Diaminobenzidine tetrahydrochloride staining, horseradish peroxidase-conjugated immunoglobulin G secondary antibodies (Table S5) were employed for 1 h at RT, and 3,3’Diaminobenzidine tetrahydrochloride agent was utilized for immunostaining. For immunofluorescence staining, sections were incubated with Alexa Fluor 488- or Alexa Fluor 594-labeled lgG secondary antibodies (Table S5) for 1 h at RT, and cell nuclei were stained with DAPI. The images were captured under a fluorescence microscope (Leica, DM3000, Germany).

### Western blots

Western blots were conducted pursuant to the method as previously described [37]. In brief, cells were harvested and lysed with RIPA lysis buffer on ice for 30 min, and they were centrifuged at 12,000 rpm to obtain the protein extract. In total, 50 μg of protein extracts were resolved on 8% to 12% sodium dodecyl sulfate-polyacrylamide gel electrophoresis gels and transferred to polyvinylidene fluoride (Millipore, Immobilon-P, IPVH00010). The membranes were blocked with QuickBlock blocking buffer (Beyotime, P0252), and they were incubated with the primary antibodies (Table S4) overnight at 4 °C. The membranes were further incubated with horseradish peroxidase-conjugated secondary antibodies (Table S5), while blots were visualized using the Efficient Chemiluminescence Kit (GENVIEW and GE2301).

### Quantitative real-time PCR and RT-PCR

Cells were lysed by TRIzol reagent (Vazyme, R401-01), and total RNA was isolated by the method in terms of the manufacturer's instructions. The cDNAs were generated using EvoM-MLVRT Premix (Accurate Biology, AG11706) according to the manufacturer's protocol using 500 ng of template RNA per reaction.

qPCR was performed by using a SYBR Green Premix Pro TaqHs qPCR kit (Accurate Biology, AG11701) on the Bio-Rad CFX96 Connect real-time PCR detection system. Data were analyzed using the 2^-ΔΔCT^ method with *GAPDH* or *ACTB* as the housekeeping gene. The primer sequences of chosen genes were shown in Table S6.

RT-PCR was conducted using 2×Taq Master Mix (Dye Plus) (Vazyme, P222-AA), and the reaction system and procedure were completed according to manufacturer's instructions. PCR products were separated by electrophoresis on a 2% agarose gel, and a Gel Documentation and Image Analysis System (ChampGel 5000) was employed to acquire the images.

### CCK-8 assay

CCK-8 (Dojindo, ck04) was utilized to detect cell proliferation of human SSC line without or with treatment of siRNAs. Cells were seeded into 96-well plates, and they were cultured for 5 d. In total, 10 μl of CCK-8 reagent were added to cells, and they were placed in an incubator for 2 to 3 h. The multimode microplate reader (Biotek, Synergy 2) was employed to measure the absorbance at 450 nm.

### EDU incorporation assay

EDU reagent A was diluted with Dulbecco's modified Eagle's medium/F12 at 1:2,000, and cells were incubated with EDU for 12 h. The cells were then fixed with 4% paraformaldehyde for 30 min and incubated with 2 mg/ml glycine for 8 min. These cells were washed with PBS containing 0.5% Triton X-100, and they were stained with the Apollo staining solution for 30 min at dark room. Finally, cells were stained with DAPI for 5 min. The EDU-positive staining was observed under a fluorescence microscope (Leica, DMi8, Germany), and the percentages of EDU-positive cells were calculated by the number of proliferating cells (EDU^+^)/the number of total cells (DAPI^+^) × 100%.

### Flow cytometry

Cell apoptosis was determined by flow cytometry using the APC Annexin V apoptosis detection kit with propidium iodide (PI) staining (Biolegend, 640932). According to the manufacturer's instructions, cells were washed with PBS twice, and then they were incubated with 5 μl of Annexin V-APC and 10 μl of PI for 15 min at RT in the dark. The numbers of Annexin V-APC-positive cells or/and PI-positive cells were measured by flow cytometry (BD Bioscience, FACSCanto II 488N).

### Cell cycle progression analysis

Cells were harvested and fixed by 70% cold ethanol overnight at −20 °C. After being washed with PBS, 50 μg/ml RNase was used to remove RNA. Then, the cell suspension was incubated with 65 μg/ml of PI for 30 min at 4 °C in the dark. The flow cytometry (BD Bioscience, Facs CantoII 488N) was utilized to detect the number of cells at G1, S, and G2/M phases.

### TUNEL assay

The TMR (red) Tunel Cell Apoptosis Detection Kit (Servicebio, G1502) was utilized to measure cell apoptosis according to the manufacturer's instructions. Cells were seeded onto 96-well plates, and dewaxing and dehydration were performed. These cells were incubated with 20 μg/ml Protease K for 10 min at RT. Next, TMR-5-dUTP Labeling Mix/TdT enzyme buffer was employed to mark apoptotic cells. Cell nuclei were stained with DAPI, and images were captured with fluorescence microscopy (Leica, DMi8, Germany).

### RNA-seq

RNA-seq and analysis were conducted by OE Biotech Co., Ltd. (Shanghai, China). Briefly, total RNA was extracted using TRIzol reagent, and RNA with excellent purity and RNA integrity was used for RNA-seq. The libraries were constructed using TruSeq Stranded mRNA LT Sample Prep Kit (Illumina, San Diego, CA, USA) in terms of the manufacturer's instructions. The libraries were sequenced on an Illumina HiSeq X Ten platform, and 150-bp paired-end reads were generated. Differential gene expression analyses were performed using the DESeq (2012) R package, while *P* <0.05 and fold change ≥2 were set as threshold for the DEGs. Hierarchical cluster analysis of DEGs was conducted to demonstrate expression pattern of genes in different groups. The GO enrichment and KEGG pathway enrichment analyses of the DEGs were performed, respectively, using R based on the hypergeometric distribution.

### Co-IP assays

Cells were lysed on ice for 30 min, and cell debris was removed by centrifugation at 12,000g for 15 min at 4 °C. Meanwhile, ^1^/_10_ supernatant was used as the input of proteins. Primary antibodies (2 μg) (Table S3) were added to cell lysate and incubated at 4 °C overnight with gentle rotation. Magnetic beads were added to proteins that were pulled down by primary antibodies, and they were incubated at RT for 1 h with gentle rotation. Beads were collected and resuspended with 500 μl of IP buffer by pipetting gently. After washes twice, elution buffer was used to harvest protein. Finally, proteins were isolated by sodium dodecyl sulfate-polyacrylamide gel electrophoresis gel, and Western blots were performed with specific antibodies in terms of the methods as mentioned above.

### Plasmid construction, protein purification, and GST pulldown

For CST-NCK2 plasmid construction, pGEX-4T1 was digested with BamH1 and EcoR1 in 37 °C for 3 h to obtain Linearized vector. The primers (F: ggatctggttcgcgtggatccATGACAGAAGAAGTTATTGTGATAGCC, R: ctcgagtcgacccgggaattcTCACTGCAGGGCCCTGACG) for the inserted fragment were designed and amplified with KOD high-fidelity enzyme. The linearized vector and PCR fragment were purified, and PCR fragments were subcloned into pGEX-4T1 by ClonExpress II One Step Cloning Kit (Vazyme, China, C112).

Plasmids were transformed into BL21 competent cells (Tsingke Biotech, China), while monoclonal cells were chosen and grown in the LB broth containing 1×ampicillin at 37 °C until reaching an *A*_600_ of 0.6 to 1.0. Protein expression was induced with 0.1 mM isopropyl-β-d-thiogalactopyranoside at 37 °C for 4 h. Cells were lysed with 20 mM tris-HCl (pH 8.0), 100 mM NaCl, 1 mM EDTA, 1% Triton X-100, and 1 mM phenylmethylsulfonyl fluoride in water. The lysate supernatant was collected by centrifugating and incubated with 0.3 ml of GST beads overnight at 4 °C, and the GST beads were washed and maintained in the mixture of glycerol and lysate.

For GST pulldown, human SSCs were lysed on ice for 30 min, and cell debris was removed by centrifugation at 12,000 g for 20 min at 4 °C. The lysate was precleared with GST beads for 30 min at 4 °C and then incubated with GST fusion proteins immobilized to GST beads overnight at 4 °C. Meanwhile, ^1^/_10_ lysate was used as the input of protein.

### IP-MS

IP-MS and analyses were conducted by OE Biotech Co., Ltd. (Shanghai, China). Briefly, antigen–antibody complex immobilized to magnetic beads was collected. After enzymatic hydrolysis and peptide desalination, the eluents containing polypeptides were collected for liquid chromatography-mass spectrometry analyses by Q Exactive Mass Spectrometer (ThermoFisher Scientific).

### Xenotransplantation of human SSCs

Xenotransplantation of human SSCs was carried out pursuant to the approach as described previously [38]. Briefly, mice (5 to 6 wk old) were injected intraperitoneally with 40 mg/kg of busulfan to remove male germ cells. After 3.5 weeks of busulfan treatment, testes were checked for the depletion of male germ cells. If no male germ cell remained in the seminiferous tubules, transplantation of the human SSC line without or with treatment of siRNAs was conducted. In total, 10 μl of cell suspension (10^7^ cells/100 μl) were transplanted into the seminiferous tubules via the rete testes. After 2 months of cell transplantation, testes were collected for histological and morphological examination.

### Statistical analysis

Statistical analyses were performed by the GraphPad Prism 6.0, and SPSS 20.0 was utilized for risk analysis of mutation sites. Each experiment was replicated at least 3 times, and all data were presented as the means ± SD. The *t* test was employed to calculate the statistical difference between the 2 groups, while chi-square test and logistic regression were used for risk analysis. *P* < 0.05 was considered the statistical significance.

## Data Availability

The data could be available with consent of the corresponding author.
