# Supplementary material for: OIP5 Interacts with NCK2 to Mediate Human Spermatogonial Stem Cell Self-Renewal and Apoptosis through Cell Cyclins and Cycle Progression and Its Abnormality Is Correlated with Male Infertility
Source: Research (Wash D C). 2023 Jun 7;6:0162. doi: 10.34133/research.0162 (PMC10246317; doi:10.34133/research.0162)
Supplement: Supplementary 1 — Figs. S1 to S8 Tables S1 to S6 [file research.0162.f1.pdf]

OIP5 interacts with NCK2 to mediate human spermatogonial stem cell self-renewal and apoptosis through cell Cyclins and cycle progression and its abnormality is correlated with male infertility

Yinghong Cui[1], Wei Chen[1], Li Du[1], Zuping He[1, 2*]

Supplemental Data

Supplemental Figures 1-8

Figure S1. The expression of OIP5 in testis and male germ cells.

Figure S2. The identification and characterization of human SSC line.

Figure S3. The validation of OIP5 siRNA knockdown efficiency.

Figure S4. Overexpression of OIP5 stimulated proliferation of human SSC line and inhibited their apoptosis.

Figure S5. Immunochemical staining showed the colocalization of SV40 (red fluorescence) with PCNA (green fluorescence) in mouse testes transplanted with human SSC line with control siRNA or OIP5 siRNA3.

Figure S6. the secondary mass spectrogram of NCK2 pulldown by OIP5.

Figure S7. The validation of NCK2 siRNA knock down efficiency.

Figure S8. The validation of NCK2 shRNA knock down efficiency.

Supplemental Tables 1-6

Table S1. Sequences of shRNAs.

Table S2. *OIP5* variants identified in NOA patients.

Table S3. Sequences of siRNAs.

Table S4. Primary antibody used in this study.

Table S5. Secondary antibodies utilized in this study.

Table S6. Primers of genes for PCR and qPCR.

Supplemental Figures

Figure S1

A

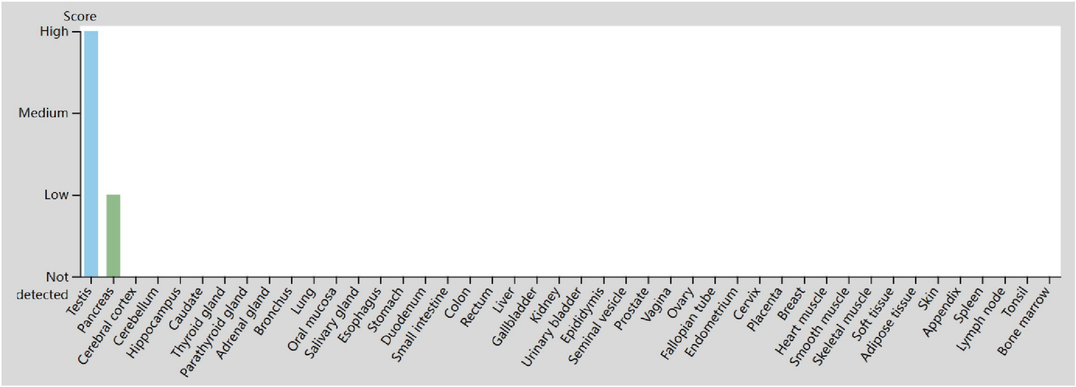

B

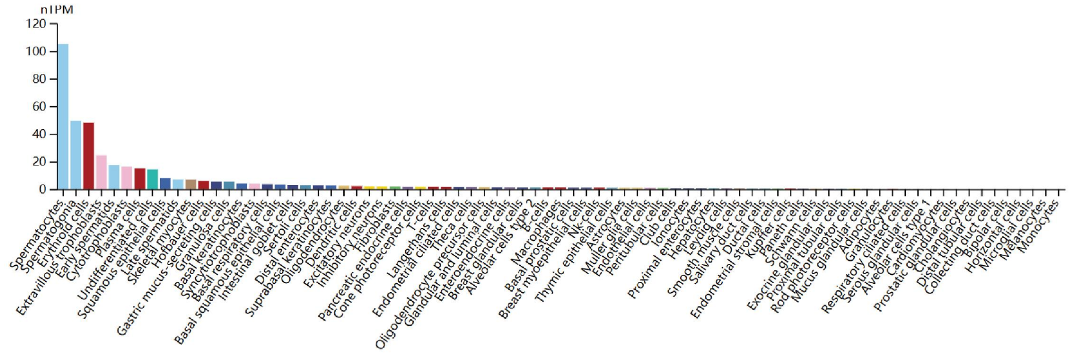

Figure S1. The expression of OIP5 in testis and male germ cells. (A) The human protein atlas database was used to analyze the OIP5 protein expression in human tissues. (B) The human protein atlas database was used to analyze the *OIP5* mRNA expression in different cells.

Figure S2

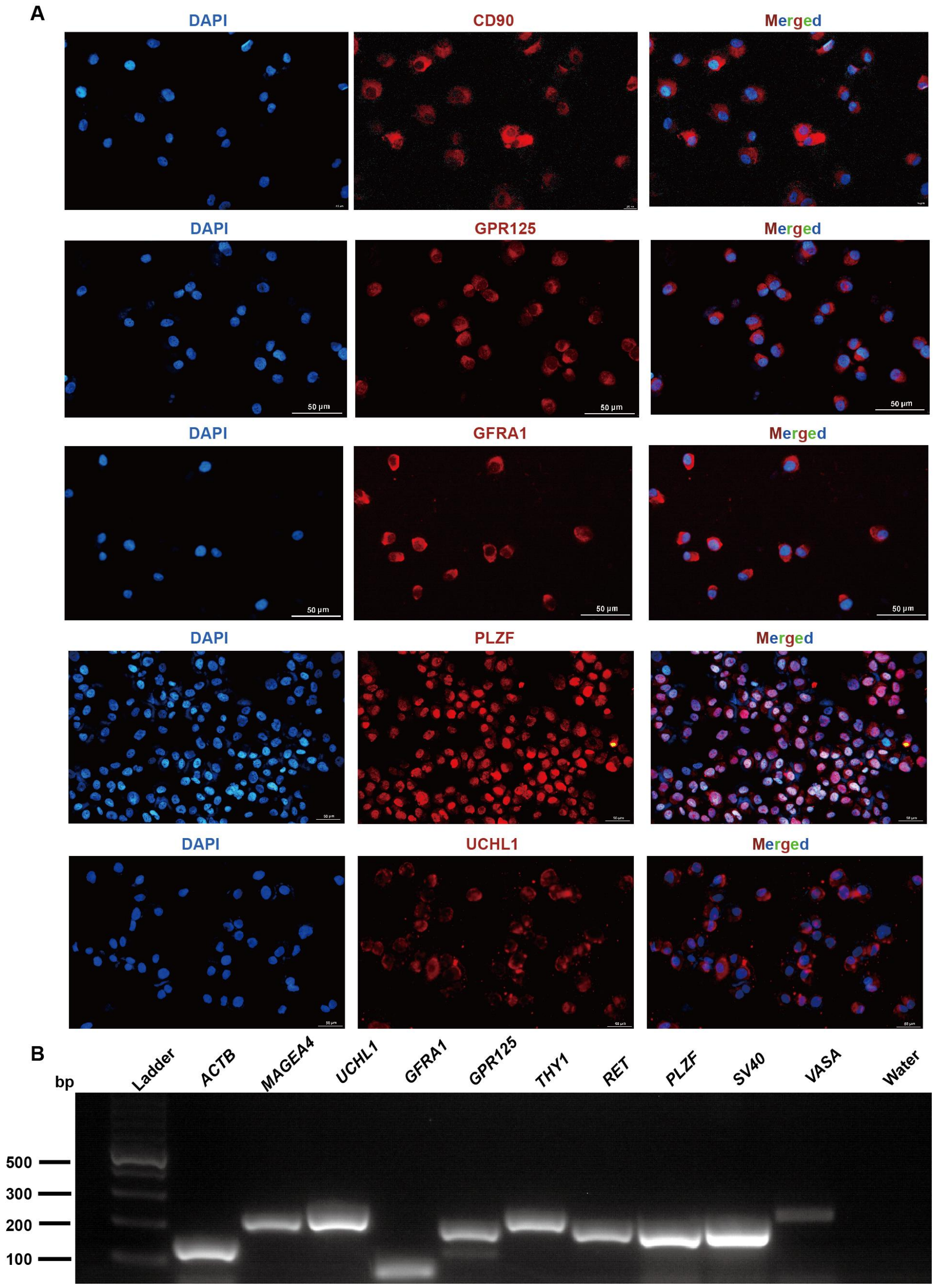

Figure S2. The identification and characterization of human SSC line. (A) Immunofluorescence showed that human SSC line expressed the proteins CD90 (THY1), GPR125, GFRA1, PLZF, and UCHL1. (B) RT-PCR displayed that human SSC line expressed transcripts of *MAGEA4*, *UCHL1*, *GFRA1*, *GPR125*, *THY1*, *RET*, *PLZF*, *SV40*, and *VASA*.

Figure S3

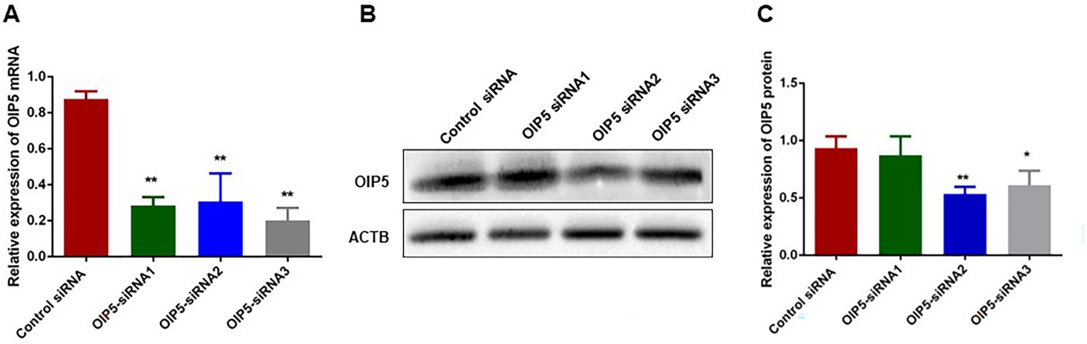

Figure S3. The validation of OIP5 siRNA knockdown efficiency. (A) qRT-PCR assay showed *OIP5* transcription in human SSC line with treatment of control siRNA and OIP5 siRNAs. (B and C) Western blots revealed OIP5 protein levels in human SSC line with treatment of control siRNA and OIP5 siRNAs. * indicated $P<0.05$, and ** denoted $P<0.01$.

Figure S4

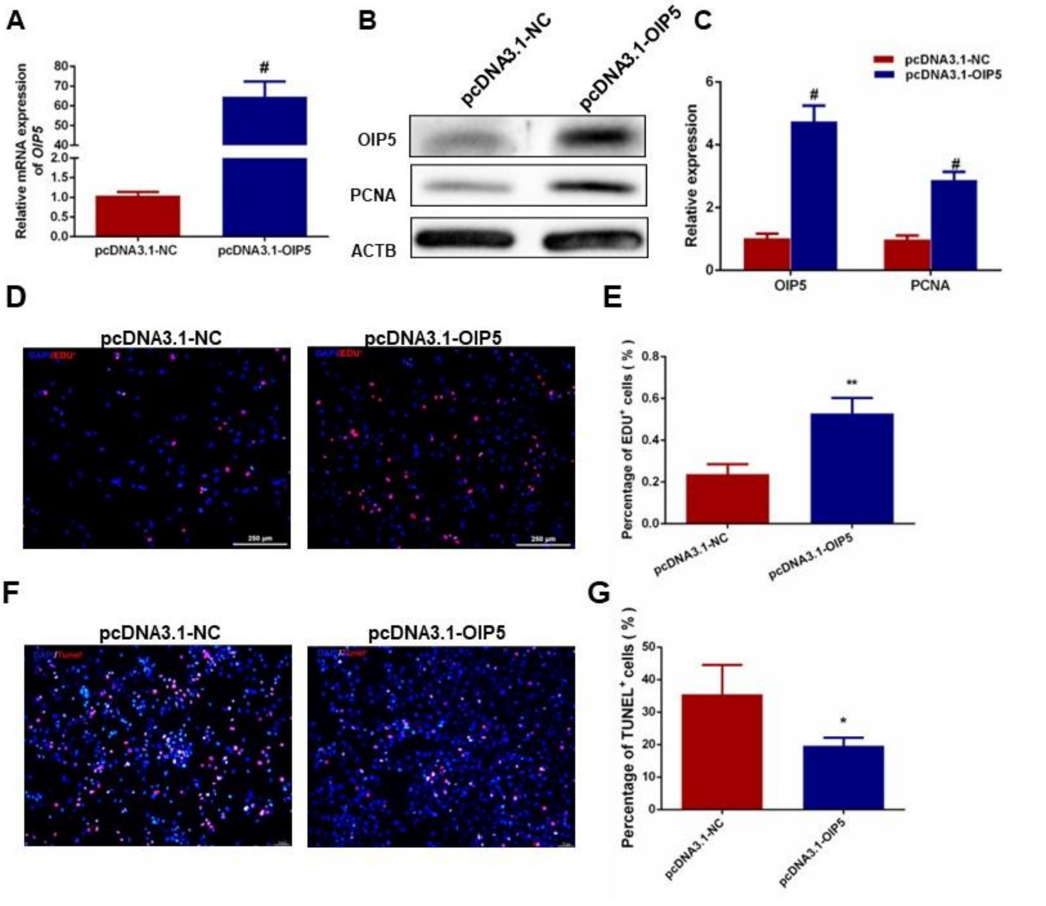

Figure S4. Overexpression of OIP5 stimulated the proliferation of human SSC line and inhibited their apoptosis. (A) qRT-PCR assay showed *OIP5* transcription level in human SSC line with OIP5 overexpression. (B and C) Western blots revealed OIP5 protein level in human SSC line with OIP5 overexpression. (D and E) The percentages of EDU-positive cells in human SSC line transfected with the pcDNA3.1-OIP5 or pcDNA3.1-NC. (F and G) The percentages of TUNEL-positive cells in human SSC line transfected with the pcDNA3.1-OIP5 or pcDNA3.1-NC. * indicated $P<0.05$, and ** denoted $P<0.01$.

Figure S5

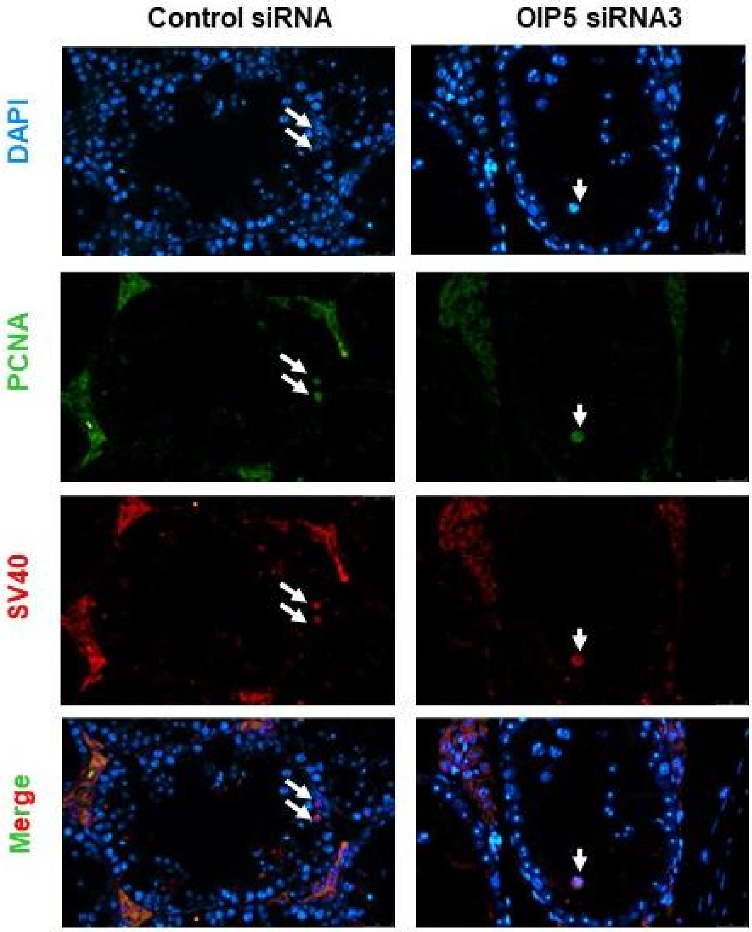

Figure S5. Immunochemical staining showed the colocalization of SV40 (red fluorescence) with PCNA (green fluorescence) in mouse testes transplanted with human SSC line with control siRNA or OIP5 siRNA3.

Figure S6

A

[K].VQLVDNVYCIGQRR.[F]

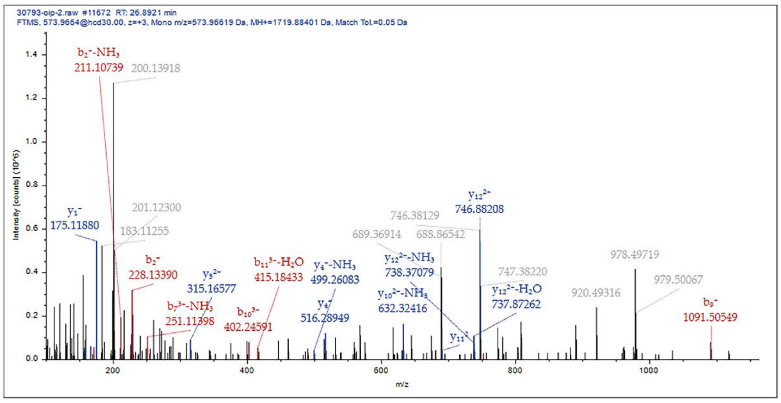

B [R].KGASLSNGQGSR.[V]

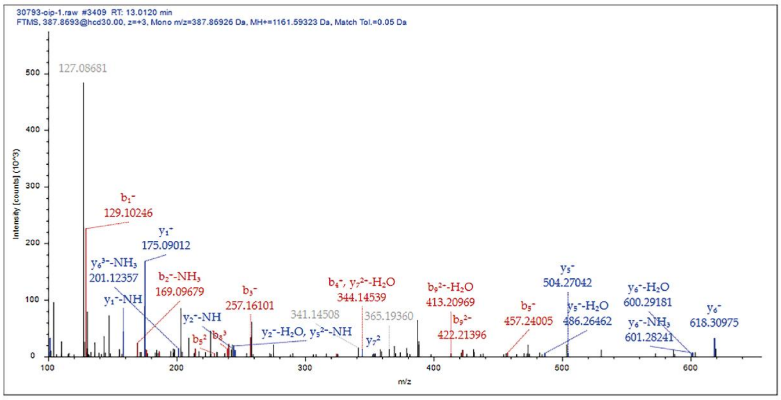

C

[K].GSLVKNLKDTLGLGK.[T]

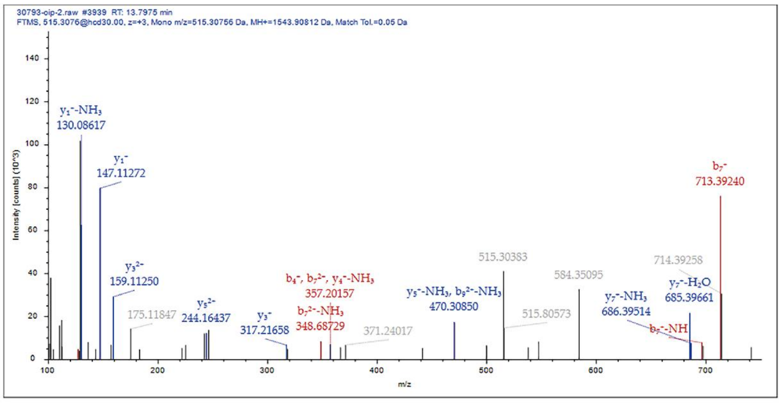

Figure S6. the secondary mass spectrogram of NCK2 pulldown by OIP5.

Figure S7

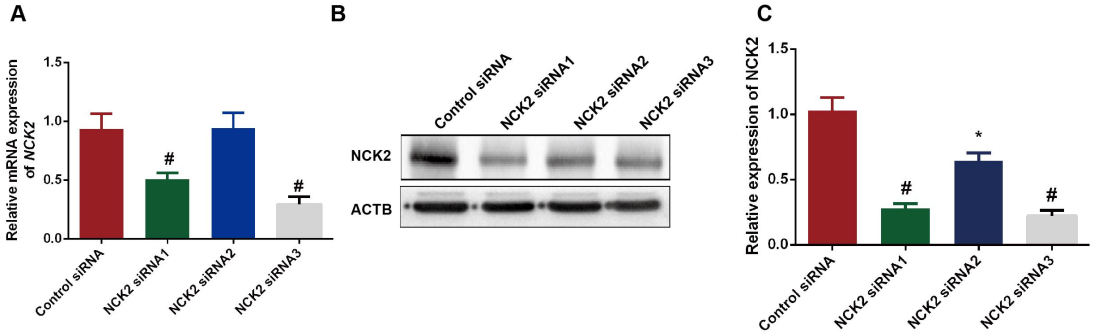

Figure S7. The validation of NCK2 siRNA knockdown efficiency. (A) qRT-PCR assay showed *NCK2* transcription in human SSC line with treatment of control siRNA and NCK2 siRNA1-3. (B and C) Western blots revealed NCK2 protein level in human SSC line with treatment of control siRNA and NCK2 siRNA1-3. * indicated $P<0.05$, and # denoted $P<0.01$.

Figure S8

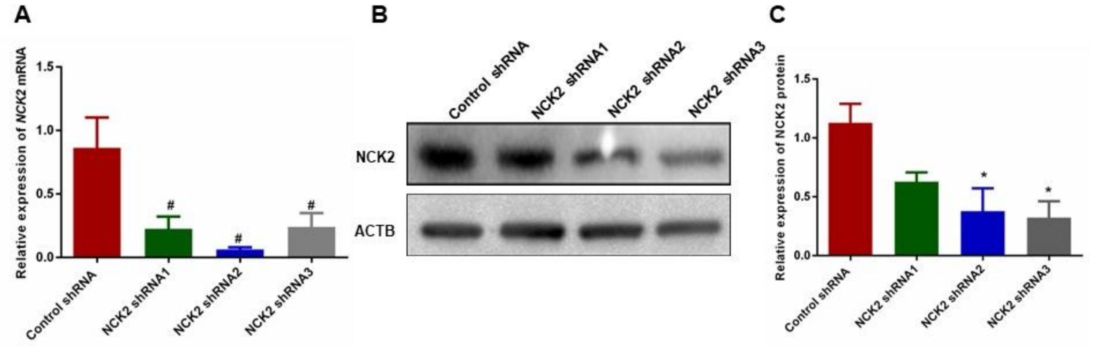

Figure S8. The validation of NCK2 shRNAs knock down efficiency. (A) qRT-PCR assay showed *NCK2* transcription in human SSC line with treatment of control shRNA and NCK2 shRNAs. (B and C) Western blots revealed NCK2 protein level in human SSC line with treatment of control shRNA and NCK2 shRNA1-3. * indicated $P<0.05$, and # denoted $P<0.01$.

Supplemental Tables 1-6

Table S1. Sequences of NCK2 shRNAs

ID	5’	stem	loop	stem	3’
NCK2-RNAi(105363-2)-a	Ccgg	cgTGGACAATGTCTACTGCAT	CTCGAG	ATGCAGTAGACATTGTCCACG	TTTTTg
NCK2-RNAi(105363-2)-b	aattcaaaaa	cgTGGACAATGTCTACTGCAT	CTCGAG	ATGCAGTAGACATTGTCCACG	
NCK2-RNAi(105364-1)-a	Ccgg	cgGGCTATGTACCGTCCAACT	CTCGAG	AGTTGGACGGTACATAGCCCG	TTTTTg
NCK2-RNAi(105364-1)-b	aattcaaaaa	cgGGCTATGTACCGTCCAACT	CTCGAG	AGTTGGACGGTACATAGCCCG	
NCK2-RNAi(105365-1)-a	Ccgg	gcTTGTTTGAATCTCACAATT	CTCGAG	AATTGTGAGATTCAAACAAGC	TTTTTg
NCK2-RNAi(105365-1)-b	aattcaaaaa	gcTTGTTTGAATCTCACAATT	CTCGAG	AATTGTGAGATTCAAACAAGC	

Table S2. ***OIP5*** **variants identified in NOA patients**

**Variation and frequency in NOA patients**						
Nucleotide change	c.G658T	c. C494T	c.T110C	c.T164G	c.G77A	c.595-8C>T
Amino acid change	p.V220L	p.S165F	p.M37T	p.L55R	p.R26K	splicing
Frequency	2/777	30/777	1/777	19/777	1/777	1/777
Variant type	heterozygous	29 heterozygous, 1 homozygous	heterozygous	heterozygous	heterozygous	heterozygous

**Allele frequency in human population**						
1000 Genomes Project	0.0004	0.023	0.0004	NA	NA	NA
East Asians in gnomAD	0.002	0.065	0.000	NA	NA	NA
All individuals in gnomAD	0.000059	0.004	0.000032	NA	NA	NA
**Functional prediction**						
GenoCanyon	Damaging (1.000)	Tolerated (0.012)	Damaging (1.000)	Damaging (1.000)	Damaging (1.000)	NA
fitCons	Damaging (0.732)	Damaging (0.706)	Tolerable (0.442)	Tolerable (0.442)	Tolerable (0.442)	NA
Mutation Assessors	Low (1.845)	Neutral (0.295)	Low (1.63)	Low (1.445)	Neutral (-0.285)	NA
ClinPred	Benign (0.01)	Benign (0.006)	Pathogenic (0.763)	Benign (0.426)	Benign (0.04)	NA
**NCBI reference sequence number of *OIP5* is GenBank: NM_007280.2; NA, not available.**						

Table S3. Sequences of siRNAs

siRNAs	Sequences (5'-3')
OIP5 siRNA -146(OIP5 siRNA1)	GGGAUACGCAGGUGGUGAATT<br>UUCACCACCUGCGUAUCCCTT
OIP5 siRNA -375(OIP5 siRNA2)	GGAAGCGCCCUUCCUAGUUTT<br>AACUAGGAAGGGCGCUUCCTT
OIP5 siRNA -468(OIP5 siRNA3)	CCAUCUGUAUUCUACCCAUTT<br>AUGGGUAGAAUACAGAUGGTT
NCK2 siRNA -642(NCK2 siRNA1)	CCCUCGUGAAGAACCUGAATT<br>UUCAGGUUCUUCACGAGGGTT
NCK2 siRNA -750(NCK2 siRNA2)	GCAUCUACGACCUCAACAUTT<br>AUGUUGAGGUCGUAGAUGCTT
NCK2 siRNA -1056(NCK2 siRNA3)	CCGAGGAGGAGCUCAACUUTT<br>AAGUUGAGCUCCUCCUCGGTT

Table S4. Primary antibody used in this study

Antibodies	Cat. No.#	Companies	Assays	Host
OIP5	HPA059602	sigma	IF, IHC	Rabbit
OIP5	12142-1-AP	proteintech	WB, IP, IHC, IF, ELISA	Rabbit
OIP5	orb477944	Biorbyt	ELISA, ICC, IHC-P, WB	Rabbit
NCK2	10206-1-AP	proteintech	IHC, WB, ELISA	Rabbit
CD90	LS-C45431	LifeSpan BioSciences	FCM, IF, IHC-fr, IP	Mouse
UCHL1	13179S	CST	WB, IHC, IF, F	Rabbit
PLZF	ab104854	abcam	WB, ICC/IF, Flow Cyt	Mouse
HumNuc	ab191181	abcam	ICC, Flow Cyt, IHC-Fr	Mouse
GPR125	ab51705	abcam	ICC/IF, ELISA	Rabbit
GFRA1	sc-10716	Santa cruz	ICC/IF	Rabbit
PCNA	ab29	abcam	IHC-P, WB, ICC/IF, Flow Cyt	Mouse
PCNA	13110s	CST	WB, IP, IHC, IF, F	Rabbit
Cyclin A2	4656T	CST	WB	Mouse
Cyclin B1	4138T	CST	WB, IF	Rabbit
Cyclin D1	2978T	CST	WB, IHC	Rabbit
Cyclin E1	4129T	CST	WB	Mouse
Cyclin H	2927T	CST	WB, IP	Rabbit
β-actin	3700S	CST	WB, IHC, IF, F	Mouse
GAPDH	2118S	CST	WB, IHC, IF, F	Rabbit

Table S5. Secondary antibodies utilized in this study

Antibodies	Companies	cat#	Reactivity
Goat Anti-Mouse HRP	Beyotime Biotechnology	A0126	Mouse
Goat Anti-Rabbit HRP	Beyotime Biotechnology	A0208	Rabbit
Alexa Fluor 488	Thermo scientific	A21202	Mouse
Alexa Fluor 555	Thermo scientific	A31572	Rabbit
Alexa Fluor 555	Thermo scientific	A31570	Mouse
Alexa Fluor 488	Thermo scientific	A21206	Rabbit

Table S6. Primers of genes for PCR and qPCR

Primer	Sequence (5'-3')	Product(bp)
ACTB-F	CACCATTGGCAATGAGCGGTTC	135
ACTB-R	AGGTCTTTGCGGATGTCCACGT	
MAGEA4-F:	CTTACCCACTACCATCAGCTTC	212
MAGEA4-R:	TGATGACTCTCTCCAGCATTTC	
UCHL1-F	AGCTGAAGGGACAAGAAGTTAG	265
UCHL1-R	TTGTCATCTACCCGACATTGG	
GFRA1-F2:	GACTCCTGCAAGACGAATTACA	87
GFRA1-R2:	GCTGCTGACAGACCTTGACT	
GPR125-F2	GCGTCATTACGGTCTTTGGAA	199
GPR125-R2	ACGGCAATTCAAGCGGAGG	
THY1-F:	CAGAAGGTGACCAGCCTAAC	233
THY1-R:	TTGCTAGTGAAGGCGGATAAG	
RET-F	TTGCCCAGATCGGGAAAGTC	206
RET-R	GGCCACCACCATGTAGTGAA	
PLZF-F	GGACAAGGTTGAGGAAAGAGG	205
PLZF-R	CAACACGGAGTAGATGCCCAG	
VASA-F:	ACTGGTCGTTGTGGGAATAC	255
VASA-R:	GGGAGCTCGTGAAGAAGAAA	
NCK2-F	CTTCTCTGCGAGTCTCTCTTTATG	160
NCK2-R	CCACTTCCGATGGAAAAGGAATAATG	
GAPDH-F:	AATCCCATCACCATCTTCC	382
GAPDH-R:	CATCACGCCACAGTTTCC	
OIP5-F	TGAGAGGGCGATTGACCAAG	189
OIP5-R	AGCACTGCGTGACACTGTG	
NCK2-F	CTTCTCTGCGAGTCTCTCTTTATG	160
NCK2-R	CCACTTCCGATGGAAAAGGAATAATG	
CCND1-F	GCCCTCGGTGTCCTACTTCAAATG	111
CCND1-R	TCCTCCTCGCACTTCTGTTCCTC	
NEDD1-F	TTAATGTGAATGCTGCTAGTGGAGGAG	89
NEDD1-R	TTGTGGTAGAACTGTGGCAATGGAC	
SPRY2-F	GCACTCGCAGGTCCATTCTTCTG	103
SPRY2-R	GCTGCTGCTGGTACTTGTCCTC	
ABCC9-F	TCTGCGTCCGGGTTTGAAAT	144
ABCC9-R	TGATTTAGCAATGGTCACAAACAA	
